# Psychometric Validation of the Turkish ORTO-11 and Psychological Correlates of Orthorexic Tendencies in Transgender Adults Before Gender-Affirming Treatment

**DOI:** 10.3390/bs16081423

**Published:** 2026-08-19

**Authors:** Caner Yeşiloğlu, Ali Meriç Kurt, Lut Tamam, Mehmet Emin Demirkol, Zeynep Namlı, Mahmut Onur Karaytuğ, Sinem Çetin Demirtaş

**Affiliations:** 1 Department of Psychiatry, Faculty of Medicine, Çukurova University, 01330 Adana, Turkey; 2 Department of Psychiatry, Gaziantep Nizip State Hospital, 27700 Gaziantep, Turkey

**Keywords:** orthorexia nervosa, ORTO-11, transgender adults, gender affirmation, health anxiety, psychological pain, self-compassion, perceived social support

## Abstract

Orthorexic tendencies and their measurement remain understudied in transgender adults. This cross-sectional study conducted a preliminary psychometric evaluation of the Turkish ORTO-11 and examined psychological correlates of orthorexic tendencies in a clinically recruited sample of transgender adults undergoing psychiatric evaluation before gender-affirming hormonal or surgical treatment. Eighty participants completed the ORTO-11, Health Anxiety Inventory, Psychache Scale, Self-Compassion Scale, and Multidimensional Scale of Perceived Social Support. Internal consistency analyses, confirmatory factor analysis, Spearman correlations, robust regression, and indirect-effect analysis were conducted. The ORTO-11 showed good internal consistency (Cronbach’s α = 0.88; McDonald’s ω = 0.89), and confirmatory factor analysis supported a unidimensional structure with acceptable model fit indices. Lower ORTO-11 scores, indicating greater orthorexic tendencies, were associated with higher health anxiety and psychological pain and with lower self-compassion and perceived social support. In multivariable analysis, psychological pain was the only significant independent correlate of ORTO-11 scores. Bootstrap analysis suggested that psychological pain statistically accounted for part of the association between health anxiety and orthorexic tendencies. These findings provide preliminary support for the Turkish ORTO-11 as a self-report measure of orthorexic tendencies in this clinically recruited transgender sample, but the scale should not be interpreted as a diagnostic instrument for orthorexia nervosa.

## 1. Introduction

In recent years, healthy eating has become a prominent topic in both public health and popular culture. In some individuals, however, a preoccupation with healthy eating may become rigid, excessive, and distressing, a phenomenon commonly referred to as orthorexia nervosa (ON) (Bratman & Knight, 2000). Orthorexic tendencies are generally characterized by an excessive focus on the quality, purity, and perceived healthfulness of food, together with restrictive food rules and avoidance of foods considered unhealthy or impure (Brytek-Matera, 2012). However, ON remains a debated construct. It is not formally recognized as an independent psychiatric disorder, and ongoing discussions concern whether it represents a distinct clinical entity, a variant of eating disorders, an obsessive–compulsive phenomenon, or a maladaptive form of health-related dietary control (Chaki et al., 2013; Donini et al., 2022). Importantly, ON is not currently recognized as an independent psychiatric diagnosis in the DSM-5 or ICD-11, and there is no universally accepted diagnostic threshold for distinguishing clinically relevant ON from non-pathological interest in healthy eating. This is particularly relevant because ORTO-based measures have been criticized for low specificity, including a tendency to classify culturally valued or health-oriented eating behaviours as pathological. In addition, previous studies have used different ORTO versions and cut-off points, and findings regarding the factor structure of ORTO-based instruments have been inconsistent, with both unidimensional and multidimensional solutions reported across cultural adaptations. Cross-cultural differences in the meaning of “healthy eating” may also influence how ORTO items are interpreted. Therefore, in the present study, we use the term “orthorexic tendencies” rather than “orthorexia nervosa diagnosis.” (Dunn & Bratman, 2016; Missbach et al., 2015; Roncero et al., 2017; Barrada & Roncero, 2018; Niedzielski & Kaźmierczak-Wojtaś, 2021).

Transgender and gender-diverse individuals have been reported to experience higher rates of eating-related symptoms and body image concerns than cisgender populations (Parker & Harriger, 2020; Rasmussen et al., 2023). These disparities can be understood within the minority stress framework, which posits that distal stressors (e.g., discrimination, victimization, and stigma) contribute to proximal stressors, including internalized stigma, expectations of rejection, and heightened vigilance, ultimately increasing psychological distress (Brooks, 1981; Meyer, 1995; Testa et al., 2015). When body image concerns intersect with gender affirmation goals, such stress may also increase vulnerability to disturbed eating behaviors. Gender-affirming body ideals, including desires for muscularity, thinness, or body congruence, may shape eating attitudes and behaviors in ways that overlap with orthorexic tendencies (Jones et al., 2016; Parker & Harriger, 2020). Emerging evidence further suggests that minority stress may contribute to disordered eating through psychological mechanisms such as body shame, negative affect, and maladaptive coping strategies (Santoniccolo & Rollè, 2024). Within this framework, orthorexic tendencies may represent attempts to regain a sense of control over health, body, or emotional distress through rigid dietary regulation. Accordingly, we hypothesized that psychological pain would statistically account for part of the association between health anxiety and orthorexic tendencies in this clinically recruited transgender sample.

Health anxiety may be particularly relevant to orthorexic tendencies because ON often involves rigid attempts to control perceived health threats through food selection and dietary rules (Koven & Abry, 2015). Previous studies have linked health anxiety with orthorexic symptoms and dietary preoccupation. Individuals with heightened health-related concerns may use restrictive or highly controlled eating patterns as a way to manage perceived vulnerability (Chace & Kluck, 2022; Greville-Harris et al., 2022). In transgender adults undergoing gender affirmation, health-related concerns may also occur in the context of medical evaluations, hormonal treatment planning, surgical expectations, and heightened attention to bodily change. These factors make health anxiety a clinically meaningful correlate to examine in relation to orthorexic tendencies (Chace & Kluck, 2022; Greville-Harris et al., 2022; Koven & Abry, 2015).

Psychological pain, or psychache, refers to intense emotional suffering associated with shame, helplessness, unmet needs, and perceived deprivation of emotional support (Holden et al., 2001). Psychological pain has been linked to maladaptive coping strategies, including disturbed eating behaviors (Tomba et al., 2021; Özbay et al., 2025). In the context of transgender adults, psychological pain may be relevant because experiences of stigma, social rejection, body incongruence, or uncertainty during gender affirmation may increase emotional distress (Parker & Harriger, 2020; Testa et al., 2015; Veale et al., 2017).

Individuals with adequate social support tend to maintain more balanced attitudes toward healthy eating, preventing such attitudes from developing into ON tendencies (Plichta & Stroebele-Benschop, 2026). In contrast, insufficient social support has been associated with disrupted eating patterns and identified as a risk factor for the development of anxiety and eating disorders (Kim et al., 2023; Wonderlich-Tierney & Vander Wal, 2010). It has been shown that individuals with lower levels of social support exhibit more problematic trajectories in eating behaviors (Stern et al., 2023). These findings suggest that social support not only serves as a determinant of psychological well-being but also acts as a protective factor against ON (Plichta & Stroebele-Benschop, 2026; Stern et al., 2023).

High self-compassion is linked to flexible eating and lower restrictive tendencies (Braun et al., 2016; Schoenefeld & Webb, 2013). Self-judgment, the tendency to avoid criticism due to body shape, and heightened perceptions of internal threat may contribute to the emergence of orthorexic behaviors (Kalika et al., 2023). In contrast, adopting a compassionate stance toward one’s thoughts and emotions facilitates the regulation of negative affect and mitigates ON symptoms (Braun et al., 2016; Kalika et al., 2022, 2023; Schoenefeld & Webb, 2013). Therefore, self-compassion is considered a psychological protective factor against ON (Adams & Leary, 2007; Kalika et al., 2022, 2023).

Among the commonly used measures to assess ON tendencies are the ORTO-15, Eating Habits Questionnaire (EHQ), Düsseldorf Orthorexia Scale (DOS), Barcelona Orthorexia Scale (BOS), and Orthorexia Nervosa Inventory (ONI) (Niedzielski & Kaźmierczak-Wojtaś, 2021). The ORTO-15 is a widely used instrument, validated in multiple languages including Turkish, German, Spanish, and Hungarian (Arusoğlu et al., 2008; Donini et al., 2005; Missbach et al., 2015; Parra-Fernández et al., 2018; Varga et al., 2014). Following criticism regarding its factor structure and internal consistency, shortened versions of the scale (e.g., an 11-item form) have been employed in numerous studies (Arusoğlu et al., 2008; Parra-Fernández et al., 2018). Although these instruments have been used in various languages and samples to assess orthorexic tendencies, their psychometric properties have not been specifically examined in gender-minority populations, including transgender adults. Psychometric properties may differ in transgender populations because responses to eating-related questionnaire items can be influenced by gender dysphoria, body congruence concerns, gender affirmation goals, and minority stress; these factors may alter item interpretation and response patterns compared with cisgender populations, underscoring the importance of validating psychometric instruments within transgender-specific clinical samples (Duffy et al., 2021; Jones et al., 2016; Linsenmeyer et al., 2024; Parker & Harriger, 2020; Testa et al., 2015). Given these conceptual and psychometric concerns, the ORTO-11 should not be considered a diagnostic instrument for ON (Dunn & Bratman, 2016; Missbach et al., 2015; Roncero et al., 2017). Rather, in this study, it was used as a measure of orthorexic tendencies, reflecting the degree of rigid preoccupation with perceived healthy eating and food-related control. This cautious interpretation is particularly relevant in transgender adults, for whom eating behaviours may be shaped by body congruence concerns, gender affirmation goals, minority stress, and broader psychosocial context (Jones et al., 2016; Parker & Harriger, 2020; Santoniccolo & Rollè, 2024; Testa et al., 2015).

Accordingly, the present study had two aims. The first aim was to examine preliminary psychometric properties of the Turkish ORTO-11 in a clinically recruited sample of transgender adults receiving gender-affirming care. The second aim was to explore associations between orthorexic tendencies and health anxiety, psychological pain, self-compassion, and perceived social support. We hypothesized that the Turkish ORTO-11 would show acceptable internal consistency and preliminary support for a unidimensional structure; greater orthorexic tendencies, reflected by lower ORTO-11 scores, would be associated with higher health anxiety and psychological pain and with lower self-compassion and perceived social support; and psychological pain would statistically account for part of the association between health anxiety and orthorexic tendencies.

## 2. Materials and Methods

### 2.1. Sample

Participants were recruited from individuals who consecutively applied to the Psychiatry Outpatient Clinic of Çukurova University Faculty of Medicine Hospital for psychiatric evaluation related to the gender affirmation process. In the present study, the “gender affirmation process” referred to an official clinical application for gender-affirming care that required psychiatric assessment as part of the clinical pathway. All eligible individuals who applied during the study period were invited to participate.

Although participants could be at different administrative or clinical stages of the gender affirmation process, none had initiated gender-affirming hormone therapy or undergone gender-affirming medical or surgical interventions at the time of assessment. Therefore, the sample represented a clinically recruited group of transgender adults evaluated before gender-affirming hormonal or surgical treatment.

The inclusion criteria were being between 18 and 65 years of age, being a transgender individual who had officially applied for the gender affirmation process, and being literate in Turkish. The exclusion criteria were the presence of an acute or clinically unstable psychiatric condition that made completion of self-report scales unsafe or unreliable, current acute substance intoxication or withdrawal, inability to provide reliable self-report data because of acute agitation, impaired reality testing, attention problems, or impaired judgment, or having a severe or uncontrolled chronic medical condition that could interfere with participation or psychometric assessment. A past history of psychiatric disorder was not an exclusion criterion if the individual was clinically stable at the time of assessment. Similarly, psychiatric medication use was not an exclusion criterion in clinically stable individuals.

Between 25 December 2024 and 25 August 2025, 87 individuals who applied for psychiatric evaluation related to the gender affirmation process were assessed for eligibility. Two individuals were excluded because of incomplete questionnaire responses, and five were excluded due to acute or clinically unstable psychiatric conditions, including one participant with a current psychotic episode, three with severe depressive episodes, and one experiencing substance withdrawal. The final sample consisted of 80 participants. Written informed consent was obtained from all participants, and all data were collected through face-to-face clinical interviews and self-report psychometric scales.

Face-to-face clinical interviews were conducted by psychiatrists experienced in the psychiatric assessment of transgender adults. No additional study-specific standardized training was provided because all interviewers routinely perform these assessments as part of their clinical practice. To ensure consistency, all interviewers followed a predefined study protocol developed under the supervision of a senior psychiatrist.

### 2.2. Procedure

In Turkey, psychiatric evaluation is a standard and mandatory component of the clinical pathway for individuals who officially apply for gender-affirming care. Therefore, presentation to the psychiatry outpatient clinic in this study did not necessarily indicate that participants were seeking treatment for a psychiatric disorder or that they were under routine psychiatric follow-up. Rather, participants were individuals who presented for a single clinical evaluation as part of the institutional gender affirmation process.

All applicants were evaluated face-to-face by psychiatry physicians using a standardized procedure. The assessment included confirmation of eligibility criteria, collection of sociodemographic and clinical information, review of psychiatric history and current medication use, and assessment of current psychiatric status. The Structured Clinical Interview for DSM-5 (SCID-5) was used to evaluate current psychiatric disorders.

Psychological distress was not considered an exclusion criterion by itself. Participants with health anxiety, psychological pain, or other forms of psychological distress were eligible if they were able to provide informed consent and complete the self-report scales safely and reliably. Individuals were excluded only when they had an acute or clinically unstable psychiatric condition that could compromise safe participation or the reliability of self-report data.

### 2.3. Sociodemographic Data Form

A structured sociodemographic information form developed by the researchers was used in this study. A clinician-administered sociodemographic form captured age, assigned sex at birth, education, substance use, BMI, and medical history. Gender identity was asked directly to participants in an open-ended format during the sociodemographic assessment. Responses were then categorized descriptively as trans man, trans woman, non-binary, or other self-described gender identity.

Information on relationship status, place of residence, smoking habits, and alcohol consumption was collected to characterize the sociodemographic and lifestyle profile of the study sample. These variables were reported descriptively and were not included in the primary analyses because they were not part of the predefined study hypotheses.

### 2.4. ORTO-11 Scale

The ORTO-15, developed by Donini et al. (2005) to assess orthorexia-related eating behaviors, is a 15-item self-report instrument. The scale evaluates individuals’ thoughts and behaviors regarding the selection, purchasing, preparation, and consumption of foods they consider healthy. Each item has four response options (always, often, sometimes, and never) and is scored from 1 to 4 according to the item-specific scoring key, with lower total scores indicating greater orthorexic tendencies (Donini et al., 2005). In the Turkish adaptation study, Arusoğlu et al. retained 11 items with factor loadings of 0.50 or higher to form the ORTO-11 and reported a Cronbach’s alpha coefficient of 0.62. This version comprises items 3, 4, 5, 6, 7, 8, 10, 11, 12, 13, and 14 of the original ORTO-15, with item 8 reverse-scored (Arusoğlu et al., 2008).

### 2.5. Health Anxiety Inventory

The Health Anxiety Inventory is an 18-item self-report measure assessing health-related thoughts, feelings, and behaviors. The first 14 items assess current health anxiety, whereas the final four assess the anticipated negative consequences of having a serious illness (Salkovskis et al., 2002). Each item is scored from 0 to 3, with higher total scores indicating greater health anxiety. In the Turkish validity and reliability study, the Cronbach’s alpha coefficient was reported as 0.91 (Aydemir et al., 2013).

### 2.6. Psychache Scale (PS)

The PS is a 13-item self-report instrument developed to assess psychological pain (Holden et al., 2001). Items are rated on a 5-point response scale, yielding total scores ranging from 13 to 65, with higher scores indicating more severe psychological pain. In the Turkish validity and reliability study, Demirkol et al. (2018) reported a Cronbach’s alpha coefficient of 0.98.

### 2.7. Self-Compassion Scale (SCS)

The SCS is a 26-item self-report scale developed by Neff (2003) to assess self-compassion. Items are rated on a 5-point Likert scale, and the scale comprises six dimensions: self-kindness, self-judgment, common humanity, isolation, mindfulness, and over-identification. Items belonging to the negatively oriented dimensions of self-judgment, isolation, and over-identification are reverse-scored when calculating the overall score, with higher scores indicating greater self-compassion. The Turkish adaptation was conducted by Akın et al. (2007), who supported the original six-factor structure and reported internal consistency coefficients ranging from 0.72 to 0.80 across the subscales.

### 2.8. Multidimensional Scale of Perceived Social Support (MSPSS)

The MSPSS is a 12-item self-report instrument assessing perceived social support from family, friends, and a significant other. Each item is rated from 1 (very strongly disagree) to 7 (very strongly agree), with higher scores indicating greater perceived social support (Zimet et al., 1988). The psychometric properties of the revised Turkish form were examined by Eker et al. (2001), who reported a Cronbach’s alpha coefficient of 0.89 for the total scale.

### 2.9. Statistical Analysis

Data analyses were conducted using IBM SPSS Statistics version 26.0 (IBM Corp., Armonk, NY, USA). Confirmatory factor analysis (CFA) was conducted using IBM SPSS Amos 26. Continuous variables were summarized using means and standard deviations or medians and interquartile ranges, depending on distributional characteristics. Categorical variables were summarized using frequencies and percentages. Normality was assessed using the Shapiro–Wilk test, visual inspection of histograms and Q–Q plots, and skewness–kurtosis values.

The internal consistency of the ORTO-11 was evaluated using Cronbach’s alpha, corrected item–total correlations, Cronbach’s alpha if item deleted, and McDonald’s omega. Internal consistency coefficients were also calculated for the Health Anxiety Inventory, Psychache Scale, Self-Compassion Scale, and Multidimensional Scale of Perceived Social Support in the present sample.

CFA was conducted to examine whether the previously proposed unidimensional structure of the Turkish ORTO-11 was acceptable in this clinically recruited transgender sample. A unidimensional model was specified a priori because the Turkish ORTO-11 was originally developed as a single-score instrument. Although the initial Turkish adaptation explored a multidimensional solution, the second and third factors contained few items and demonstrated inadequate internal consistency. Consequently, the authors recommended interpreting the scale using a single total score (Arusoğlu et al., 2008). The present study therefore aimed to evaluate the fit of this previously proposed unidimensional structure rather than to re-examine alternative factorial models. CFA was performed in Amos using maximum likelihood estimation, with ORTO-11 items treated as continuous indicators. Model fit was evaluated using the chi-square statistic, chi-square/degrees of freedom ratio, comparative fit index (CFI), normed fit index (NFI), goodness-of-fit index (GFI), root mean square error of approximation (RMSEA), and standardized root mean square residual (SRMR).

The sample size was also evaluated with reference to recommended practices for confirmatory factor analysis. With 80 participants and ORTO-11 items, the present study included approximately 7.3 participants per item, which is consistent with commonly recommended participant-to-item ratios for preliminary psychometric validation studies (Brown, 2015; Kline, 2016). Because the sample size was modest and the ORTO-11 items were ordinal Likert-type indicators, CFA findings were interpreted as preliminary evidence rather than definitive confirmation of the scale structure.

Associations among study variables were examined using Spearman correlation analyses because several variables deviated from normal distribution. To reduce the risk of type I error due to multiple correlation testing, the Benjamini–Hochberg false discovery rate correction was applied to the correlation matrix.

Multiple linear regression analysis with HC3 heteroskedasticity-robust standard errors was conducted to examine variables independently associated with ORTO-11 total scores. Health anxiety, psychological pain, self-compassion, perceived social support, age, and years of education were entered as independent variables. Multicollinearity was assessed using variance inflation factor and tolerance values. Regression assumptions were examined using residual plots, Q–Q plots of standardized residuals, standardized residual values, leverage values, and Cook’s distance.

An indirect-effect analysis was performed using bootstrapping procedures with 5000 resamples to examine whether psychological pain statistically accounted for part of the association between health anxiety and ORTO-11 scores. Bias-corrected 95% confidence intervals were estimated for the indirect effect. Given the cross-sectional design, this analysis was interpreted as an exploratory indirect association and not as evidence of a causal mediation pathway.

Group comparisons according to assigned sex at birth (assigned female at birth [AFAB] vs. assigned male at birth [AMAB]) were conducted using the Mann–Whitney U test for continuous variables and Fisher’s exact test for categorical variables, as appropriate. Effect sizes for Mann–Whitney U tests were calculated using r (Z/√N). Because of the modest sample size and small subgroup sizes, formal measurement invariance testing across AFAB and AMAB participants was not conducted; therefore, subgroup comparisons were interpreted cautiously. A two-tailed significance level of *p* < 0.05 was used for statistical analyses.

Relationship status, place of residence, smoking status, and alcohol use were collected and reported to describe the sociodemographic and health-behavioural context of the clinically recruited sample. These variables were not included in the primary multivariable model because the study was primarily designed as a preliminary psychometric validation and exploratory correlate study, the sample size was modest, and some variables showed limited variability, particularly place of residence. Therefore, inferential analyses focused on the pre-specified psychological variables and core demographic covariates, namely age and years of education. Smoking and alcohol use were interpreted as contextual health-related behaviours rather than as primary explanatory variables.

## 3. Results

The final sample consisted of 80 transgender adults receiving gender-affirming care. As shown in Table 1, the median age was 27 years, the median duration of education was 16 years, and the median BMI was 25.0 kg/m^2^. The sample included 42 participants assigned female at birth (AFAB) and 38 participants assigned male at birth (AMAB). Fifty participants reported having a partner, 47 reported smoking, 42 reported social alcohol use, and 7 reported a history of substance use. Descriptive statistics for the main psychometric variables are also presented in Table 1. Regarding self-identified gender identity, all participants reported a binary transgender identity. The sample included 42 trans men, all of whom were assigned female at birth, and 38 trans women, all of whom were assigned male at birth. No participant identified as non-binary or reported another self-described gender identity.

The internal consistency and factorial structure findings for the ORTO-11 are summarized in Table 2. The ORTO-11 demonstrated good internal consistency in the present sample, with Cronbach’s α = 0.882 and McDonald’s ω = 0.885. Corrected item–total correlations ranged from 0.408 to 0.754, and Cronbach’s alpha if item deleted ranged from 0.860 to 0.885, indicating that no item substantially reduced internal consistency. Detailed item-level internal consistency statistics are provided in Supplementary Table S1.

Internal consistency estimates for the other scales were also acceptable to excellent in the present sample: Health Anxiety Inventory, α = 0.925, ω = 0.927; Psychache Scale, α = 0.980, ω = 0.980; Self-Compassion Scale, α = 0.964, ω = 0.964; and Multidimensional Scale of Perceived Social Support, α = 0.941, ω = 0.942.

**Figure 1:**
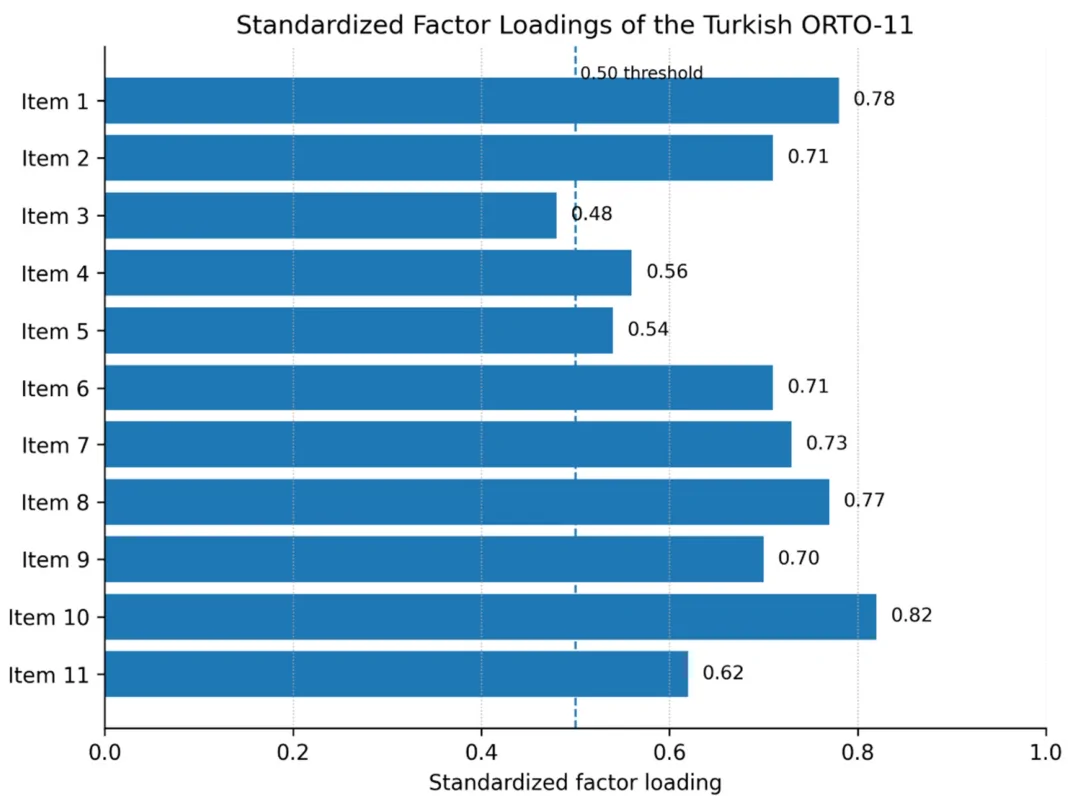
Standardized factor loadings of the Turkish ORTO-11 items. The dashed vertical line indicates the conventional 0.50 threshold.

As presented in Table 2, confirmatory factor analysis provided preliminary support for a unidimensional structure of the Turkish ORTO-11. The single-factor model demonstrated generally acceptable model fit (χ^2^ = 53.76, χ^2^/df = 1.22, *p* = 0.149, CFI = 0.969, NFI = 0.856, GFI = 0.894, RMSEA = 0.053, SRMR = 0.059). Although the CFI indicated good model fit and the SRMR was within the acceptable range, the NFI and GFI were slightly below the conventional 0.90 threshold, and the RMSEA was marginally above the criterion commonly considered indicative of close fit (<0.05). Item-level examination showed that ORTO-11 Item 3 had the lowest standardized factor loading (λ = 0.48). Although this loading was slightly below the conventional 0.50 threshold, the item was retained because its corrected item–total correlation was acceptable (0.408), and deleting the item produced only a negligible increase in Cronbach’s alpha from 0.882 to 0.885. Therefore, retaining Item 3 was considered appropriate to preserve the original item composition and facilitate comparison with previous Turkish ORTO-11 studies (Hair et al., 2019). Standardized factor loadings for each ORTO-11 item are presented in Figure 1.

As a supplementary dimensionality check, exploratory factor analysis indicated adequate sampling suitability and a predominantly unidimensional structure. These exploratory results are reported in Supplementary Table S2.

Spearman correlation analyses are presented in Table 3. Lower ORTO-11 scores, indicating greater orthorexic tendencies, were significantly associated with higher health anxiety, higher psychological pain, lower self-compassion, and lower perceived social support. These associations remained significant after Benjamini–Hochberg false discovery rate correction.

Specifically, ORTO-11 scores were negatively correlated with health anxiety (ρ = −0.568, FDR-adjusted *p* < 0.001) and psychological pain (ρ = −0.681, FDR-adjusted *p* < 0.001), and positively correlated with self-compassion (ρ = 0.673, FDR-adjusted *p* < 0.001) and perceived social support (ρ = 0.652, FDR-adjusted *p* < 0.001). ORTO-11 scores were also weakly associated with years of education (ρ = −0.294, FDR-adjusted *p* = 0.021). The weak association between ORTO-11 scores and age did not remain significant after FDR correction.

The multivariable regression model is summarized in Table 4. Multiple linear regression analysis with HC3 heteroskedasticity-robust standard errors showed that the model explained 59.4% of the variance in ORTO-11 score, *p* < 0.001. Among the independent variables, psychological pain was the only statistically significant independent correlate of ORTO-11 scores, B = −0.166, β = −0.309, *p* < 0.001. Health anxiety, self-compassion, perceived social support, age, and years of education were not independently associated with ORTO-11 scores in the multivariable model. As also shown in Table 4, multicollinearity diagnostics did not indicate severe multicollinearity. All VIF values were below 3.0, and tolerance values were above 0.36.

The mediation analysis is presented in Table 5. Health anxiety was positively associated with psychological pain, B = 0.670, *p* < 0.001, and psychological pain was negatively associated with ORTO-11 scores, B = −0.281, *p* < 0.001.

The total relationship of health anxiety on ORTO-11 scores was significant, B = −0.372, *p* < 0.001. After psychological pain was assessed in the model, the direct relationship remained significant but was reduced in magnitude, B = −0.184, *p* = 0.005. The bootstrapped indirect effect was significant, B = −0.188, 95% bias-corrected CI [−0.280, −0.111]. These findings suggest that psychological pain statistically accounted for part of the association between health anxiety and orthorexic tendencies.

AFAB and AMAB participants did not differ significantly in BMI, ORTO-11 scores, health anxiety, psychological pain, self-compassion, or perceived social support. These comparisons are provided in Supplementary Table S3.

## 4. Discussion

The prevalence of eating disorders is higher in transgender populations compared to cisgender individuals (Rasmussen et al., 2025). In transgender adolescents and young adults, the SCOFF Questionnaire is a valid tool for screening eating disorders, and the Eating Disorder Examination Questionnaire Short Form (EDE-QS) has demonstrated measurement and construct validity (Duffy et al., 2021; Linsenmeyer et al., 2024). However, to the best of our knowledge, the psychometric properties of an orthorexia-related self-report measure have not previously been examined in transgender adults. The principal psychometric finding of the present study was that the ORTO-11 showed good internal consistency and preliminary support for the proposed unidimensional structure in this clinically recruited transgender sample.

In our study, the Cronbach’s alpha coefficient was 0.88, indicating good internal consistency in this clinically recruited transgender sample. Direct comparison of item functioning between the present sample and the sample included in the original Turkish ORTO-11 adaptation study was not possible because that study did not report confirmatory factor analysis indices or item-level measurement invariance analyses. However, the internal consistency observed in the present sample (Cronbach’s α = 0.882) was higher than that reported in the original Turkish ORTO-11 study (Cronbach’s α = 0.62), while the present CFA findings provided preliminary support for the use of a single total score in this clinically recruited transgender sample (Arusoğlu et al., 2008). Nevertheless, because measurement invariance or differential item functioning analyses require direct comparison groups, future studies should examine whether ORTO-11 items function similarly across transgender and cisgender Turkish samples. For comparison, the Spanish ORTO-11-ES study reported a Cronbach’s alpha of 0.79 (Parra-Fernández et al., 2018), and the Polish validation study of the revised six-item ORTO-R reported a Cronbach’s alpha of 0.82 (Brytek-Matera et al., 2023). The internal consistency coefficient observed in the present sample was within the conventionally acceptable range (George & Mallery, 2010).

Arusoğlu et al. tested a three-factor structure in the Turkish adaptation of the ORTO-11. However, due to the small number of items in the second and third factors and the low internal consistency coefficients of these subfactors, the structure was deemed statistically inadequate. The clustering of the majority of items under a single factor rendered the single-factor model of the Turkish ORTO-11 statistically more meaningful. However, item-level findings should also be interpreted cautiously. ORTO-11 Item 3 demonstrated the lowest standardized factor loading (λ = 0.48), slightly below the conventional 0.50 threshold. This relatively lower loading indicates that Item 3 had a weaker association with the underlying construct in this clinically recruited transgender sample. Eating-related attitudes and behaviours in transgender individuals may be influenced by gender-related body concerns and minority stress, which could potentially affect responses to eating-related questionnaire items (Parker & Harriger, 2020; Noebel et al., 2023; Testa et al., 2015). Parra-Fernández et al., in the ORTO-11-ES study, tested both single- and three-factor structures and reported satisfactory fit for both models. In the Polish validation study of the ORTO-R, a revised six-item version of the ORTO-15 was used, and this structure was supported in terms of construct validity and internal consistency (Brytek-Matera et al., 2023). In our study, the unidimensional CFA solution was consistent with the use of a single total ORTO-11 score in this clinically recruited transgender sample. This analytic approach was selected because it was consistent with the intended scoring procedure of the Turkish ORTO-11 and with the recommendations of its original validation study rather than with the multidimensional structure proposed for the original ORTO-15.

Despite these encouraging psychometric findings, the present results should be interpreted in light of the inherent limitations of the ORTO-11. Although the instrument has been widely used to assess orthorexic tendencies, it has not demonstrated sufficient validity for clinical diagnosis and should not be interpreted as a diagnostic tool for orthorexia nervosa. Moreover, no universally accepted cut-off score has been established to distinguish clinically significant orthorexia from a non-pathological interest in healthy eating (Dunn & Bratman, 2016). Furthermore, previous studies have questioned the specificity of ORTO-based measures, suggesting that they may classify culturally accepted or health-oriented eating behaviours as pathological (Missbach et al., 2015; Barrada & Roncero, 2018). Therefore, the findings of the present study should be interpreted as reflecting variation in orthorexic tendencies rather than evidence of a formal psychiatric disorder. Future studies should evaluate newer instruments with improved psychometric properties, such as the ORTO-R, in transgender populations (Brytek-Matera et al., 2023). Accordingly, the main contribution of the present study is the preliminary psychometric validation of the ORTO-11 as a self-report measure of orthorexic tendencies in a clinically recruited transgender sample, rather than its validation for the clinical diagnosis of orthorexia nervosa.

Descriptively, the median ORTO-11 score in the present sample was 26.0 (IQR = 21.0–31.0). This value was close to those reported in Turkish samples not specifically recruited on the basis of transgender status. In the original Turkish validation study conducted among academic and administrative university personnel, the mean ORTO-11 score was 26.85 ± 4.40 among participants who were not undergoing dietary treatment (Arusoğlu et al., 2008). Similarly, Dolapoglu et al. (2023) reported a median ORTO-11 score of 25.5 (range = 15–40) in a nonclinical sample of Turkish medical students. Within the subgroup classified as having high orthorexic tendencies, Dolapoglu et al. also observed a weak inverse correlation between ORTO-11 and health anxiety scores (ρ = −0.242, *p* = 0.035). This association was directionally consistent with the correlation observed in our full sample (ρ = −0.568, FDR-adjusted *p* < 0.001), although their magnitudes should not be directly compared because the analyses were conducted in differently defined samples. These findings place the ORTO-11 scores in our sample within a broadly similar descriptive range. However, differences in recruitment, sample composition, summary statistics, and analytic approach—and the absence of a concurrent cisgender Turkish comparison group—preclude conclusions about whether orthorexic tendencies are more or less prevalent among transgender adults.

Furthermore, in line with previous findings, the observed negative correlation between ORTO-11 and HAI scores provides supporting construct-related evidence for the scale. These findings suggest that orthorexic tendencies are significantly associated with health anxiety (Greville-Harris et al., 2022). The elevated risk of eating disorders among transgender individuals has been discussed in relation to psychosocial factors, including body dissatisfaction, perfectionism, anxiety symptoms, and low self-esteem (Nagata et al., 2020). While the relationship between psychiatric disorders, psychological variables, and ON has not been fully explored, some studies have begun to address this issue. For instance, Awad et al. (2021) found that depression and anxiety partially mediated the association between perseverance and ON. In the present study, psychological pain statistically accounted for part of the association between health anxiety and orthorexic tendencies. Among transgender individuals, minority stress and internalized stigma may undermine self-worth and contribute to psychological pain, which may in turn be associated with maladaptive eating patterns (McGregor et al., 2023).

Similarly, Chace and Kluck (2022) reported a moderate positive correlation between pathological orthorexia and health anxiety. Kalika et al. (2022) found that self-compassion was negatively associated with orthorexic tendencies among vegan individuals. These findings are consistent with previous studies suggesting that ON is not only linked to cognitive control over eating but also closely related to psychological distress and coping resources. The association between psychological pain intensity and orthorexic tendencies in the present transgender sample further highlights the potential relevance of broader psychological distress. Our study further suggests that higher self-compassion and greater perceived social support are associated with lower orthorexic tendencies at the bivariate level. These findings are consistent with previous literature suggesting that self-compassion and social support may function as protective psychosocial resources in relation to eating-related difficulties (Kelly & Carter, 2015; Watson et al., 2017). However, in the multivariable model, psychological pain emerged as the only significant independent correlate of ORTO-11 scores. Therefore, the associations of self-compassion and perceived social support with orthorexic tendencies should be interpreted cautiously and may partly reflect their shared variance with psychological pain and broader distress-related processes.

In our study, no statistically significant differences were found between trans men, all of whom were assigned female at birth (AFAB), and trans women, all of whom were assigned male at birth (AMAB), in terms of orthorexic tendencies, self-compassion, health anxiety, perceived social support, or psychological pain. Noebel et al. (2023) reported greater ON symptomatology among transgender women than among cisgender men and cisgender women. However, that study did not establish a direct difference between transgender women and transgender men and used a different orthorexia measure, limiting direct comparability with our findings. Budge et al. (2013) found that social support was associated with lower psychological distress and that the modeled relationships among transition status, loss, coping, social support, and distress were broadly similar for transgender women and men. The fact that our sample consisted entirely of individuals undergoing gender affirmation processes may have influenced these results; however, self-acceptance, resilience, and access to social support across different stages of gender affirmation were not directly assessed.

No significant association was observed between orthorexic tendencies and BMI. In contrast, Özenoğlu et al. (2025) reported that orthorexic tendencies increased with BMI among Turkish university students, although differences in the study populations and orthorexia measures limit direct comparison. Years of education showed a weak inverse bivariate association with ORTO-11 scores, indicating greater orthorexic tendencies among participants with more years of education. However, education was not independently associated with ORTO-11 scores in the multivariable model; therefore, this finding should be considered exploratory. Although eating behaviours among transgender individuals may be influenced by body-related and gender-affirmation-related factors (Nagata et al., 2020), these factors were not directly assessed and cannot explain the BMI or education findings observed in the present study.

The relatively high rates of smoking and alcohol use observed in this sample should not be interpreted as incompatible with orthorexic tendencies. Orthorexic tendencies primarily refer to rigid preoccupation with the perceived quality, purity, and healthfulness of food and dietary control, rather than to a globally health-promoting lifestyle across all behavioural domains (Bratman & Knight, 2000; Brytek-Matera, 2012; Koven & Abry, 2015). Consistent with this interpretation, Oberle et al. (2022) found that orthorexia symptomatology was not significantly associated with the overall level or abuse of nicotine or alcohol, although associations with selected substance-use motives were observed. Therefore, dietary preoccupation may coexist with smoking or alcohol use. Because these behaviours were assessed only categorically in the present study, their frequency, severity, motives, and relationships with ORTO-11 scores could not be determined.

Several limitations should be considered. First, the study was conducted in a single center with a relatively small, clinically recruited Turkish-speaking sample; therefore, the findings cannot be generalized to all transgender adults or to community-based transgender populations. Furthermore, the sample included only trans men and trans women and did not include nonbinary or other gender-diverse individuals. Participants were recruited from a psychiatry outpatient clinic as part of the institutional gender affirmation assessment process. Although attendance at psychiatric evaluation in this context reflected the institutional clinical pathway rather than treatment-seeking for a psychiatric disorder, this clinically recruited sample may differ from transgender individuals who do not seek, cannot access, or are not engaged in gender-affirming healthcare. Therefore, the findings should be interpreted as applying primarily to transgender adults undergoing clinical assessment before gender-affirming treatment rather than to the broader transgender population. In addition, the pre-treatment assessment process involves comprehensive clinical evaluations and close attention to health-related issues, which may increase awareness of bodily health and potentially influence responses to measures of health anxiety and eating-related attitudes. Moreover, because the present study did not include a cisgender comparison group, it was not possible to determine whether the observed associations between orthorexic tendencies, health anxiety, psychological pain, self-compassion, and perceived social support are specific to transgender adults or reflect broader psychological processes that may also occur in other populations. The absence of a concurrent comparison group also precluded measurement-invariance and differential-item-functioning analyses. Although previous studies have reported elevated rates of eating-related symptoms and body image concerns among transgender and gender-diverse individuals (Parker & Harriger, 2020; Noebel et al., 2023), the present study was not designed to test between-group differences or establish transgender-specific risk. Accordingly, our findings should be interpreted as characterizing these associations within a clinically recruited transgender sample rather than demonstrating that they are unique to transgender individuals. Second, although the CFA findings provided preliminary support for a unidimensional structure, the sample size was modest for confirmatory factor analysis of an 11-item scale. The supplementary exploratory and confirmatory factor analyses were also conducted in the same sample and therefore do not constitute independent cross-validation. In addition, test–retest reliability was not assessed, and the ORTO-11 was not compared with a clinical interview or a more recently developed orthorexia instrument. Therefore, the psychometric findings should be considered preliminary and require replication in larger and more heterogeneous samples. Third, although none of the participants had initiated gender-affirming hormone therapy or undergone gender-affirming surgical interventions, participants may still have differed in the administrative, social, or psychological stages of the gender affirmation process. These unmeasured differences may have influenced body-related concerns, health anxiety, psychological pain, and eating-related attitudes. In addition, because the study was cross-sectional, changes in orthorexic tendencies and related psychological variables across later stages of gender affirmation could not be examined, and the indirect-effect analysis cannot establish temporal ordering or a causal mediation pathway.

Future research should replicate these findings in larger and more diverse transgender and gender-diverse samples recruited from multiple clinical and community settings, including nonbinary individuals. Longitudinal studies are needed to examine how orthorexic tendencies change across different stages of the gender affirmation process, including before and after gender-affirming hormone therapy or surgical interventions. Future studies should also include concurrent cisgender comparison groups to examine whether the observed associations differ across populations or reflect broader psychological processes. Independent validation samples and test–retest assessments would provide stronger evidence regarding the stability and reproducibility of the psychometric findings. In addition, the psychometric performance of newer measures of orthorexic tendencies, such as the ORTO-R, should be evaluated in transgender populations and compared directly with the ORTO-11. Finally, cognitive interviewing and item-level analyses, including measurement invariance and differential item functioning, may help determine whether specific questionnaire items function differently across transgender, gender-diverse, and cisgender populations.

## 5. Conclusions

Our findings indicate that orthorexic tendencies, psychological pain, and health anxiety are interrelated constructs in transgender individuals. In transgender people undergoing gender affirmation, where surgical and hormonal interventions are planned, psychological assessment should be multifactorial and also address nutrition and lifestyle habits. The present findings provide evidence for the internal consistency and unidimensional factorial structure of the ORTO-11 as a self-report measure of orthorexic tendencies in clinically recruited transgender adults; however, they do not establish its diagnostic validity, and the scale should not be used as a substitute for clinical assessment or as a diagnostic tool for orthorexia nervosa. By providing initial psychometric evidence for the Turkish ORTO-11 in this population, the present study addresses an important gap in the literature, although independent replication is required.

## Figures and Tables

**Table 1:** Participant characteristics and descriptive statistics of study variables.

Variable	Total Sample (*n* = 80)
Age, median (IQR)	27.0 (24.0–29.25)
Education, years, median (IQR)	16.0 (12.0–16.0)
BMI, median (IQR)	25.0 (21.80–28.00)
Self-identified gender identity	
Trans men, all assigned female at birth, *n* (%)	42 (52.5)
Trans women, all assigned male at birth, *n* (%)	38 (47.5)
Non-binary	0
Other self-described gender identity	0
Having a partner, *n* (%)	50 (62.5)
No partner, *n* (%)	30 (37.5)
Urban residence, *n* (%)	79 (98.8)
Rural residence, *n* (%)	1 (1.2)
Smoking, *n* (%)	47 (58.8)
Alcohol use, *n* (%)	42 (52.5)
Past substance use, *n* (%)	7 (8.8)
Chronic medical condition, *n* (%)	16 (20.0)
ORTO-11, median (IQR)	26.0 (21.0–31.0)
Health Anxiety Inventory, median (IQR)	19.0 (12.0–28.0)
Psychache Scale, median (IQR)	25.0 (16.0–34.0)
Self-Compassion Scale, median (IQR)	74.5 (62.0–100.0)
MSPSS, median (IQR)	64.0 (49.25–77.0)

BMI = body mass index; IQR = interquartile range; MSPSS = Multidimensional Scale of Perceived Social Support.

**Table 2:** Internal consistency estimates and factorial structure of the Turkish ORTO-11.

Analysis/Scale	Result
Internal consistency of ORTO-11	
Cronbach’s α	0.882
McDonald’s ω	0.885
Corrected item–total correlation range	0.408–0.754
Cronbach’s α if item deleted range	0.860–0.885
Confirmatory factor analysis of ORTO-11	
χ^2^	53.76
df	44
χ^2^/df	1.22
*p*	0.149
CFI	0.969
NFI	0.856
GFI	0.894
RMSEA	0.053
SRMR	0.059
Standardized factor loading range	0.48–0.82
Error variance range	0.29–0.77

ORTO-11 = Orthorexia Nervosa Scale-11; CFI = Comparative Fit Index; NFI = Normed Fit Index; GFI = Goodness-of-Fit Index; RMSEA = Root Mean Square Error of Approximation.

**Table 3:** Spearman correlations among study variables.

	ORTO-11	HAI	PS	SCS	MSPSS	Age
HAI	−0.568 (<0.001)	—				
PS	−0.681 (<0.001)	0.526 (<0.001)	—			
SCS	0.673 (<0.001)	−0.650 (<0.001)	−0.657 (<0.001)	—		
MSPSS	0.652 (<0.001)	−0.468 (<0.001)	−0.671 (<0.001)	0.703 (<0.001)	—	
Age	0.231 (0.079)	−0.182 (0.166)	−0.140 (0.285)	0.132 (0.285)	0.057 (0.665)	—
Education	−0.294 (0.021)	0.134 (0.285)	0.250 (0.058)	−0.219 (0.095)	−0.214 (0.100)	0.036 (0.780)
BMI	0.170 (0.195)	−0.132 (0.285)	−0.143 (0.285)	0.197 (0.132)	0.231 (0.079)	0.017 (0.879)

Values are Spearman’s rho coefficients with Benjamini–Hochberg FDR-adjusted *p*-values in parentheses. ORTO-11 = Orthorexia Nervosa Scale-11; HAI = Health Anxiety Inventory; PS = Psychache Scale; SCS = Self-Compassion Scale; MSPSS = Multidimensional Scale of Perceived Social Support; BMI = body mass index.

**Table 4:** Multiple linear regression analysis of factors associated with ORTO-11 scores.

Predictor	B	Robust SE	β	*p*	95% CI for B	Tolerance	VIF
Intercept	23.258	7.129	—	0.001	9.286 to 37.229	—	—
Health Anxiety Inventory	−0.077	0.081	−0.115	0.341	−0.236 to 0.082	0.516	1.938
Psychache Scale	−0.166	0.049	−0.309	<0.001	−0.262 to −0.070	0.445	2.246
Self-Compassion Scale	0.051	0.053	0.172	0.333	−0.052 to 0.154	0.369	2.711
MSPSS	0.083	0.047	0.202	0.076	−0.009 to 0.174	0.405	2.468
Age	0.206	0.129	0.146	0.109	−0.046 to 0.458	0.932	1.073
Years of education	−0.366	0.233	−0.144	0.117	−0.822 to 0.091	0.943	1.060

Model statistics: R^2^ = 0.594, adjusted R^2^ = 0.560, F(6, 73) = 21.51, *p* < 0.001. ORTO-11 = Orthorexia Nervosa Scale-11; MSPSS = Multidimensional Scale of Perceived Social Support; B = unstandardized regression coefficient; β = standardized regression coefficient; SE = standard error; CI = confidence interval; VIF = variance inflation factor.

**Table 5:** Bootstrap-based indirect-effect analysis: Health anxiety, psychological pain, and ORTO-11 scores.

Effect	B	SE	t	*p*	95% Cl
Path a: Health Anxiety → Psychological Pain	0.670	0.120	5.596	<0.001	0.432 to 0.908
Path b: Psychological Pain → ORTO-11	−0.281	0.051	−5.483	<0.001	−0.383 to −0.179
Total effect: Health Anxiety → ORTO-11	−0.372	0.063	−5.872	<0.001	−0.499 to −0.246
Direct effect: Health Anxiety → ORTO-11	−0.184	0.064	−2.876	0.005	−0.312 to −0.057
Indirect effect via Psychological Pain	−0.188	0.043	—	—	−0.280 to −0.111

ORTO-11 = Orthorexia Nervosa Scale-11.

## Data Availability

The datasets generated and analyzed during the current study are not publicly available due to ethical restrictions but are available upon reasonable request and subject to ethics committee approval.

## Supplementary Materials

The following supporting information can be downloaded at: https://www.mdpi.com/article/10.3390/bs16081423/s1, Table S1. ORTO-11 item-level reliability statistics. Table S2. Exploratory dimensionality check of the ORTO-11. Table S3. Comparisons between AFAB and AMAB participants.

## Abbreviations

The following abbreviations are used in this manuscript:

ON	Orthorexia nervosa
ORTO-11	Orthorexia Nervosa Scale-11/Turkish ORTO-11
ORTO-15	Orthorexia Nervosa Scale-15
ORTO-11-ES	Spanish ORTO-11 Eating Scale
ORTO-R	Revised ORTO scale
EHQ	Eating Habits Questionnaire
DOS	Düsseldorf Orthorexia Scale
BOS	Barcelona Orthorexia Scale
ONI	Orthorexia Nervosa Inventory
HAI	Health Anxiety Inventory
PS	Psychache Scale
SCS	Self-Compassion Scale
MSPSS	Multidimensional Scale of Perceived Social Support
BMI	Body Mass Index
DSM-5	Diagnostic and Statistical Manual of Mental Disorders, Fifth Edition
CFA	Confirmatory Factor Analysis
EFA	Exploratory Factor Analysis
SPSS	Statistical Package for the Social Sciences
AMOS	Analysis of Moment Structures/IBM SPSS Amos
ML	Maximum Likelihood
CFI	Comparative Fit Index
NFI	Normed Fit Index
GFI	Goodness-of-Fit Index
RMSEA	Root Mean Square Error of Approximation
df	Degrees of freedom
FDR	False Discovery Rate
HC3	Heteroskedasticity-consistent standard error estimator, type 3
VIF	Variance Inflation Factor
SE	Standard Error
CI	Confidence Interval
IQR	Interquartile Range
AFAB	Assigned Female at Birth
AMAB	Assigned Male at Birth
KMO	Kaiser–Meyer–Olkin measure of sampling adequacy
EDE-Q	Eating Disorder Examination Questionnaire
EDE-QS	Eating Disorder Examination Questionnaire Short Form
SCOFF	SCOFF Questionnaire
