# Supplementary material for: Psychometric Validation of the Turkish ORTO-11 and Psychological Correlates of Orthorexic Tendencies in Transgender Adults Before Gender-Affirming Treatment
Source: Behav Sci (Basel). 2026 Aug 19;16(8):1423. doi: 10.3390/bs16081423 (PMC13509684; doi:10.3390/bs16081423)
Supplement: Supplementary file 1 [file behavsci-16-01423-s001.zip › behavsci-4371354-supplementary.pdf]

Supplementary Table S1. ORTO-11 item-level reliability statistics

ORTO-11 Item	Corrected item–total correlation	Cronbach’s α if item deleted
Item 1	0.701	0.865
Item 2	0.627	0.870
Item 3	0.408	0.885
Item 4	0.486	0.879
Item 5	0.463	0.879
Item 6	0.618	0.870
Item 7	0.662	0.867
Item 8	0.692	0.866
Item 9	0.627	0.870
Item 10	0.754	0.860
Item 11	0.535	0.876

ORTO-11 = Orthorexia Nervosa Scale-11. Corrected item–total correlations ranged from 0.408 to 0.754. Cronbach’s α if item deleted ranged from 0.860 to 0.885.

Supplementary Table S2. Exploratory dimensionality check of the ORTO-11

Component	Initial eigenvalue	% of variance	Cumulative %
1	5.167	46.97	46.97
2	0.966	8.78	55.75
3	0.937	8.52	64.27
4	0.875	7.96	72.22
5	0.673	6.12	78.35
6	0.570	5.18	83.53
7	0.469	4.26	87.79
8	0.401	3.64	91.43
9	0.376	3.42	94.86
10	0.311	2.83	97.68
11	0.255	2.32	100.00

KMO = 0.883. Bartlett's test of sphericity: $\chi^2 = 352.59$, df = 55, $p < .001$.

Supplementary Table S3. Comparisons between AFAB and AMAB participants

Variable	AFAB, median (IQR)	AMAB, median (IQR)	U	P	Effect size r
BMI	24.7 (21.67–27.34)	25.7 (22.62–28.38)	669.0	.216	−0.139
Self-Compassion Scale	81.5 (67.25–99.75)	68.0 (58.50–99.50)	931.0	.202	0.143
ORTO-11	26.5 (21.25–31.00)	25.0 (21.00–31.75)	793.5	.969	−0.005
Health Anxiety Inventory	17.0 (11.25–25.75)	22.0 (13.00–29.75)	673.5	.232	−0.134
Psychache Scale	25.5 (16.00–29.50)	23.0 (17.00–39.75)	750.5	.650	−0.051
MSPSS	66.0 (51.00–78.00)	61.0 (47.75–72.75)	874.5	.464	0.082

AFAB = assigned female at birth; AMAB = assigned male at birth; BMI = body mass index; MSPSS = Multidimensional Scale of Perceived Social Support.
